# Characterization of Liver Metastases During Catheter-Directed Liver Interventions: A Comparison between Dual Phase Cone-Beam Computed Tomography and Conventional Contrast-Enhanced Computed Tomography

**DOI:** 10.5334/jbsr.2052

**Published:** 2020-07-08

**Authors:** Geert Maleux, Maria-Louisa Izamis, Cedric Werbrouck, Alessandro Radaelli, Hans Prenen, Eric Van Cutsem, Vincent Vandecaveye

**Affiliations:** 1 Department of Radiology, University Hospitals Leuven, BE; 2 Department of Imaging and Pathology, KU Leuven, Leuven, BE; 3 Philips Healthcare, NL; 4 Department of Digestive Oncology, University Hospitals Leuven, Leuven, BE

**Keywords:** cone beam computed tomography, diagnostic efficacy, liver, metastases, radioembolization

## Abstract

**Objectives:**

To compare the diagnostic performance of intra-arterial dual phase cone-beam computed tomography (DP-CBCT) with contrast-enhanced computed tomography (CE-CT) when characterizing tumor burden in patients with metastatic liver cancer.

**Materials and Methods:**

This retrospective study included 29 patients with colorectal (n =10), breast (n = 9) and neuroendocrine (n = 10) liver metastases, referred for catheter-directed treatment. Tumor type, number, maximum size, and appearance were assessed. Paired-sample t-tests compared image quality, tumor numbers, and diameters between imaging modalities.

**Results:**

Image quality was not different between DP-CBCT and CE-CT (p = 0.9). In 18 patients (62%) DP-CBCT and CE-CT showed diffuse, uncountable metastases in the liver. Of the remaining 11 patients, DP-CBCT identified two patients with diffuse tumors that appeared as a sum of 17 distinct metastases on CE-CT. In the remaining nine patients a total of 102 metastases were found using both DP-CBCT and CE-CT. Tumor detection accuracy was 98% in DP-CBCT and 67% in CE-CT (p = 0.025). Metastases were larger in diameter on DP-CBCT: colorectal: 57 +/– 9.5 mm versus 43 +/– 8.3 mm (p = 0.02); breast: 57 +/– 10 mm versus 43 +/– 8.5 mm (p = 0.03) and neuroendocrine: 56 +/– 6.3 mm versus 51 +/– 5.8 mm (p = 0.01). Rim enhancement appeared in 100% of patients with colorectal and 89% of patients with breast metastases on DP-CBCT, but was variable on CE-CT. Neuroendocrine tumors had variable rim enhancement within the same patient and across imaging modalities.

**Conclusions:**

DP-CBCT of the liver may demonstrate larger metastatic tumor burden and lesion size with a variable contrast enhancement compared to CE-CT.

## Introduction

Metastatic liver disease is the most common cause of malignant liver tumors [1]. For the majority of patients, neither surgical resection nor local ablative therapies are options given the diffuse nature of the tumors, their unfavorable location close to major vessels or bile ducts, or the overall poor condition of the patient [2]. For these patients, chemotherapy and best supportive care are the backbone of oncologic treatment [1]. However, for liver-only or liver-dominant metastatic disease in conjunction with acceptable liver function, catheter-directed liver therapies including transarterial chemoembolization (TACE) [3,4], hepatic arterial infusion therapy (HAIT) [5], liver chemosaturation [6] or Yttrium-90 radioembolization [7] can be performed. Today, most of the catheter-directed liver interventions are performed as salvage therapy.

Although liver magnetic resonance imaging (MRI) or positron emission tomography (PET) – computed tomography (CT) have a better sensitivity for the detection of liver metastases than conventional contrast-enhanced CT (CE-CT), the latter is most frequently performed because of its high availability, speed of performance, and ability to analyze not only the liver but also lung, mediastinal, bone, peritoneal, and other distant metastases [8,9].

Over the last decade, cone-beam CT (CBCT) has been introduced as an adjunctive imaging tool during interventional liver procedures [10,11,12]. CBCT aids tumor detection and characterization, angiographic guidance, and prediction of interventional success [13,14]. While CBCT has demonstrated its value extensively in the interventional management of hepatocellular carcinoma (HCC) [10,15,16,17,18,19,20,21], there is limited evidence of its value for liver metastases. To address this need, we conducted a retrospective study comparing the diagnostic performance of intra-arterial dual phase CBCT with conventional intra-venous CE-CT to characterize tumor burden in the liver during catheter-directed liver therapies for metastatic disease.

## Materials and Methods

### Study population

This retrospective study was approved by the local ethics committee and included all patients referred to the interventional radiology department of a tertiary academic oncologic referral center for catheter-directed liver interventions between September 2013 and August 2015. The patients were discussed at the multidisciplinary tumor board, which included medical, surgical, and radiation oncologists, as well as diagnostic and interventional radiologists, pathologists, and nuclear medicine physicians. All patients gave informed consent for the angiographic procedures.

During the study time frame, 35 patients were admitted to the hospital for catheter-directed liver interventions. Three had no CE-CTs, two had failed DP-CBCTs due to motion artefacts, and one had only an arterial-phase CT. The 29 patients who had both CE-CT and DP-CBCT images were included in the study. The mean time interval between CE-CT and DP-CBCT was 28 days (min/max: 1/147 days). Nine patients had breast cancer liver metastases, 10 patients had colon cancer liver metastases, and 10 had neuroendocrine cancer liver metastases. Patient demographics, indication for liver-directed therapy and type of catheter-directed liver intervention are summarized in Table 1.

**Table 1:** Patient demographics and type of catheter-directed liver intervention.

Diagnosis	Gender		Age		Liver Intervention		

	M	F	Mean	Min-Max	Y-90	HAIT	TACE

BREAST	0	9	56	46–81	0	9	0
COLON	7	3	53	44–70	7	0	3
NEURO-ENDOCRINE	6	4	60	46–70	5	0	5

Y-90: resin-based yttrium-90 radioembolization. HAIT: hepatic artery infusion therapy with 12ml of Mitomycin C. TACE: transarterial chemoembolization with use of irinotecan-loaded microspheres.

The patients had unresectable or unablatable liver-only or liver-dominant metastases, refractory to chemotherapy with an Eastern Cooperative Oncology (ECOG) performance status of 0 or 1. Laboratory values included albumin > 2.5 mg/dL; total bilirubin < 2.0 mg/dL; international normalized ratio (INR) < 1.5; platelet count > 50.000/mm^3^; liver enzymes < 5 times upper limit.

Yttrium-90 radioembolization was performed with resin microspheres (Sirtex Inc, Cosgrove, Australia). HAIT comprised selective lobar infusion of 2 × 6 mg Mitomycine C. Chemoembolization protocols depended on the origin of the metastases: 25 mg superabsorbent polymer (SAP) microspheres (Quadraspheres, Merit Medical, South Jourdan, UT, USA) with a dry diameter of 50–100 µM and mixed with 75 mg doxorubicin/m^2^ were used for treating neuroendocrine liver metastases, while 2 ml of 100–300 µM hydrogel microspheres (LC/DC-beads BTG – Biocompatibles, Surrey, UK) mixed with 2 × 100 mg irinotecan were used for treating colorectal liver metastases.

### Conventional venous phase CT technique

Typically, the CT comprised an image taken 90 seconds after injection of 120 ml of iodized contrast medium (Visipaque 320, GE Healthcare, Machelen, Belgium) into an antebrachial vein with an injection rate of 1.5 ml/sec. Images with a section thickness of 3 mm were reconstructed every 3 mm to provide contiguous sections.

### Intraprocedural dual phase cone-beam CT technique

All interventions were performed using a flat-panel angiographic system with DP-CBCT modality (AlluraClarity FD20; Philips Healthcare, Best, the Netherlands) by one interventional radiologist (GM) with 20 years of experience in liver interventions. After local anesthesia, a 4 French (F) sheath (Boston Scientific, Natick, MA, USA) was inserted in the right common femoral artery followed by catheterization of celiac trunk and superior mesenteric artery with a 4F Simmons 1 catheter (Performa, Merit Medical, South Jourdan, UT, USA) to define the hepatic arterial anatomy and portal venous patency. Subsequently, the right and left hepatic artery were cannulated with use of different types of microcatheters (Embocath Plus, Merit Medical, South Jourdan, UT, USA; Progreat 2.7, Terumo Europe, Leuven, Belgium; Direxion 2.8, Boston Scientific, Natick, MA, USA or Cantata 2.5, Cook Medical, Bloomington, IN, USA). Digital subtraction angiography (DSA) through the microcatheter was performed for the right and left hepatic artery, followed by DP-CBCT. For the right liver lobe a total of 20 ml undiluted iodized contrast medium was injected at a rate of 2ml/second. For the left liver lobe, a total of 10 ml of iodinated contrast medium was injected at a rate of 1 ml/second. In both cases, the arterial phase scan was triggered five seconds after the start of the contrast injection, while the venous phase scan followed with an eight-second delay after the end of the first scan. The 312 two-dimensional projections acquired during each CBCT scan (120 kVp tube voltage, 50–325 mA tube current, 5.2 seconds acquisition, 240° rotation, 250 × 250 × 190 mm field-of-view) were automatically transferred to a workstation (Philips, Interventional Workspot, Best, The Netherlands) where three-dimensional (3D) reconstructions with an isotropic resolution of 0.6 mm could be viewed side-by-side for further analysis [11].

### Imaging analysis

Image analysis was performed in consensus. All CE-CT-scans were analyzed on a picture archiving and communication system (PACS) (Agfa Healthcare, Mortsel, Belgium); all DP-CBCT-scans were analyzed on the interventional workstation by two imaging specialists with 3 and 20 years of experience, respectively. First, all CE-CT were sequentially analyzed, followed by sequential analysis of all DP-CBCT-images in order to minimize potential bias. For each CE-CT the number of all metastases, the diameter of the largest metastasis and the contrast enhancement compared to the residual liver parenchyma were assessed. If more than 20 metastases were identified, the case was categorized as ‘diffuse’; the contrast enhancement of the metastases was considered hypodense, isodense, or hyperdense compared to the residual liver parenchyma. Image quality of both CE-CT and DP-CBCT was assessed separately according to the following scoring system: (1) excellent difference in contrast enhancement between tumor and liver parenchyma & sharp delineation of the metastases; (2) excellent difference in contrast enhancement between tumor and liver parenchyma and poor delineation of the metastases; (3) poor difference in contrast enhancement between tumor/liver parenchyma; (4) suboptimal image quality, which required confirmation by additional imaging; (5) inadequate/non-diagnostic. In the case of DP-CBCT imaging, it was also assessed in which phase (arterial or venous) the metastases were best visualized.

### Statistical analysis

Patient demographics (gender, age, and liver intervention) were considered categorical variables, and age was expressed with median and a range (min-max). Paired-sample *t* tests were used to compare quality, tumor count, and tumor diameters between imaging modalities. Tumor detection accuracy was determined as the fraction of tumors found by one modality compared to the total number of tumors found by both modalities.

## Results

### Image quality score

Average image quality scores did not differ significantly between CE-CT and DP-CBCT (1.48 and 1.45 respectively, p = 0.9). For both modalities, 83% of cases (n = 24) had an image quality score of 1 or 2, and no images were considered non-diagnostic (Table 2). For DP-CBCT the majority of liver metastases were best visualized in the venous phase (n = 19, 66%). In 28% of cases (n = 8) arterial was as representative as venous. In only 7% of cases (n = 2) the arterial phase was best.

**Table 2:** Image quality scores of conventional CT and cone beam CT.

Score	Interpretation	CE-CT	DP-CBCT

1	Excellent contrast between tumor and liver parenchyma & sharp delineation of the metastases;	21	23
2	Excellent contrast between tumor and liver parenchyma & poor delineation of the metastases;	3	1
3	Poor contrast tumor/liver parenchyma;	4	3
4	Suboptimal image quality which required confirmation by additional imaging;	1	2
5	Inadequate/non-diagnostic.	–	–

CE-CT: contrast enhanced computed tomography (venous phase). DP-CBCT: dual phase cone-beam computed tomography.

**Figure 1:**
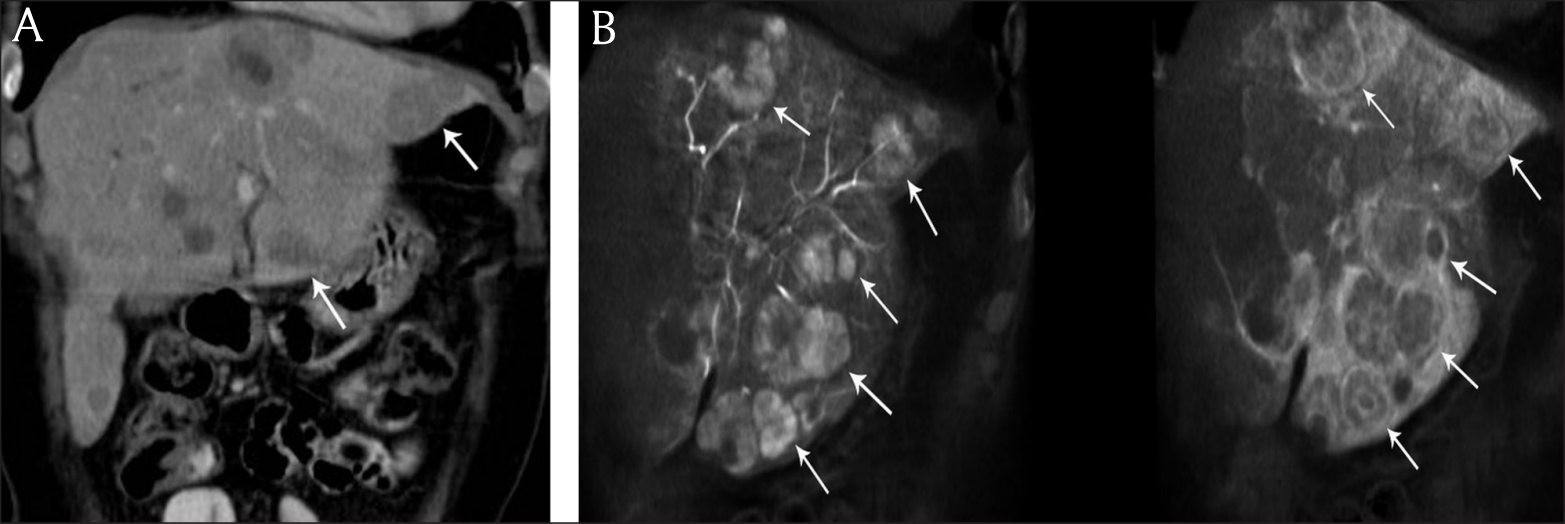
**A)** Coronal reconstructed contrast-enhanced computed tomography and **B)** cone beam computed tomography imaging in an 58-year-old woman presenting with bilobar neuroendocrine liver metastases. Increased number of liver metastases (white arrows) are identified on cone beam computed tomography imaging versus contrast-enhanced computed tomography.

**Figure 2:**
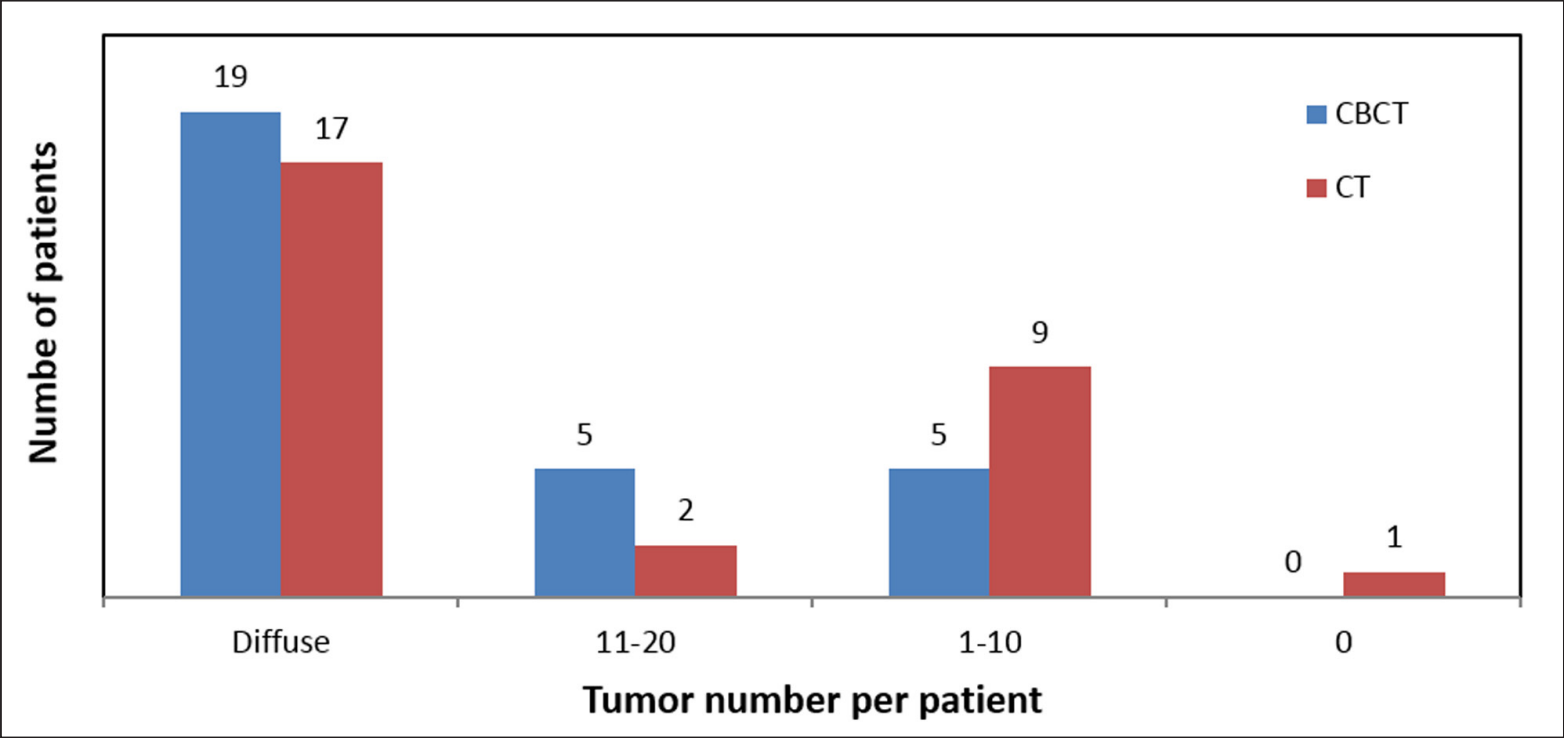
For the same cohort of patients, CBCT depicted more tumors per patient than CT.

*Tumor number*. DP-CBCT and CE-CT were in agreement on tumor number and distribution in 62% (18/29) of patients where tumors were diffuse or borderline diffuse (i.e. > 20 tumors) (Table 3). Of the remaining 11 patients, DP-CBCT identified two patients with diffuse tumors that appeared as 17 distinct metastases on CE-CT. In the other 9 out of 11 patients, a total of 102 countable metastases were found using both DP-CBCT and CE-CT with a tumor detection accuracy of 98% in DP-CBCT and 67% in CE-CT (p = 0.025) (Figures 1 and 2).

**Table 3:** Tumor detection and classification.

Diagnosis	No. tumors (diffuse: >20)		Largest tumor diameter (mm)		Density (hypo/hyper/iso)		Rim enhancement? (y/n)		Days between CE-CT and DP-CBCT

	CE-CT	DP-CBCT	CE-CT	DP-CBCT	CE-CT	DP-CBCT*	CE-CT	DP-CBCT	

BREAST	11	15	23	26	hypo	hypo	y	y	71
	20	20	39	80	hypo/hyper	hypo/hyper	n	y	1
	diffuse	diffuse	25	24	hypo	hypo	n	y	1
	diffuse	diffuse	88	95	hypo	hypo	n	y	7
	diffuse	diffuse	68	73	hypo	hypo	n	n	11
	diffuse	diffuse	17	24	hypo	hypo	mixed	y	25
	diffuse	diffuse	45	67	hypo/hyper	hypo/iso	y	y	6
	diffuse	diffuse	44	72	hypo	hypo	mixed	y	8
	diffuse	diffuse	not countable	not countable	hypo	hypo	n	y	5
COLON	0	6	not detected	15	not detected	hyper	not detected	y	16
	2	1	65	72	hypo	hypo	mixed	y	18
	4	8	30	57	hypo	hypo/hyper	n	y	28
	5	8	18	23	hypo	hyper; one hypo dense	n	y	14
	6	17	56	73	hypo	hypo	y	y	15
	7	11	35	33	hypo	hypo	y	y	15
	7	diffuse	29	40	hypo	hypo(v); hypo/hyper (a)	n	y	53
	10	diffuse	96	108	hypo	hypo	n	y	82
	10	11	33	82	hypo	hypo	n	y	147
	diffuse	diffuse	27	29	hypo	hypo	n	y	33
NEURO-ENDOCRINE	4	3	90	103	hypo/hyper	hypo/hyper	y	y	53
	diffuse	diffuse	73	68	hypo	hypo	n	n	8
	diffuse	diffuse	54	54	hypo	hyper	n	y	85
	diffuse	diffuse	50	52	hypo/iso	hyper (v); hypo/iso (a)	n	y	39
	diffuse	diffuse	40	44	hypo	hypo/iso	n	y	–1
	diffuse	diffuse	30	34	hypo/iso	hyper	n	n	3
	diffuse	diffuse	41	47	hyper/hypo	hypo	y	y (a)	18
	diffuse	diffuse	31	36	hypo (a); iso (v)	hypo(v); hyper (a)	n	y	91
	diffuse	diffuse	53	62	iso/hypo	hypo(v); hyper (a)	y	y	1
	diffuse	diffuse	46	56	hypo	hypo	y	y	6

* venous phase. hypo: hypodense. hyper: hyperdense. iso: isodense.

**Figure 3:**
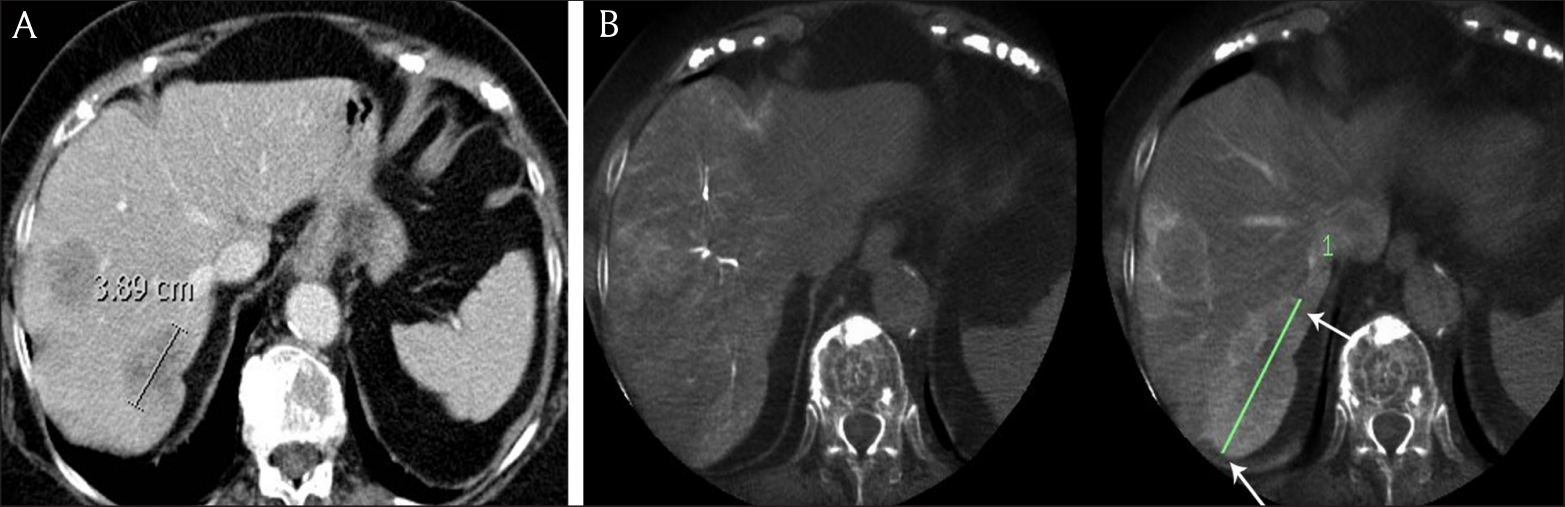
**A)** Contrast-enhanced computed tomography and **B)** cone beam computed tomography in an 81-year-old female patient with breast cancer liver metastases demonstrates the longest diameter of the largest metastasis (white arrows), measuring respectively 3.9 cm and 7.9 cm.

**Figure 4:**
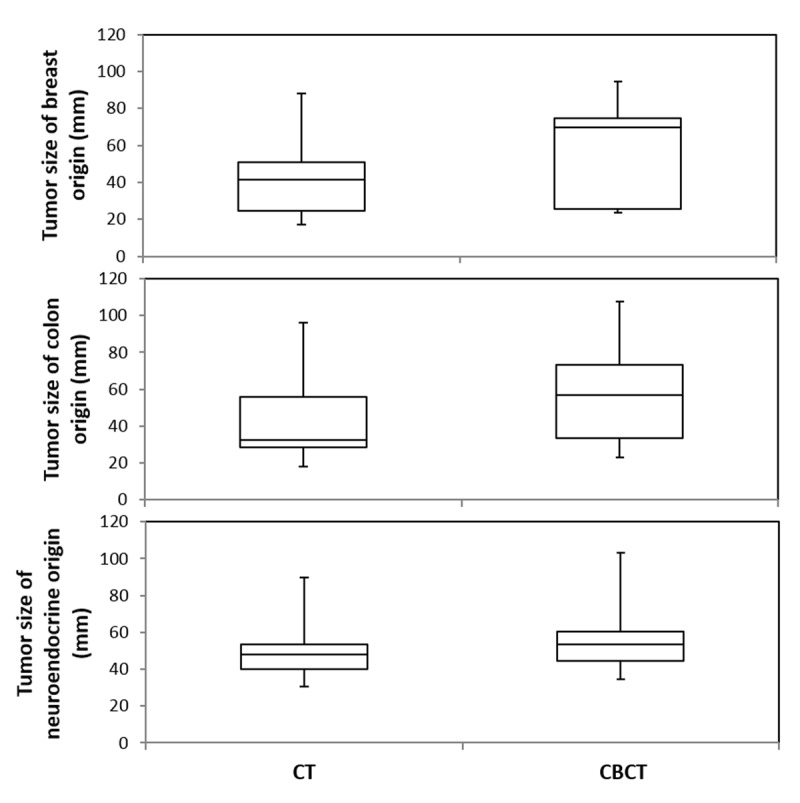
DP-CBCT detected significantly larger tumor diameters compared to CE-CT, regardless of the tumor origin (breast, p = 0.03; colon, p = 0.03; neuroendocrine, p = 0.01). Box and whisker plots of the maximum tumor diameter where, for each plot, the top-most value of the whisker represents the maximum tumor diameter, followed by the third quartile range (top box), median value (at division of boxes), first quartile range (bottom box), and minimum value at bottom-most end of whisker. The distribution of values for tumors of breast and colon origin is equally different between CBCT and CT; there is a smaller difference amongst tumors of neuroendocrine origin.

*Tumor size*. Tumors consistently appeared significantly larger on DP-CBCT than on CE-CT regardless of origin: breast: 57 ± 10mm versus 43 ± 8.5mm (p = 0.03) (Figure 3); colon: 57 ± 9.5mm versus 43 ± 8.3mm (p = 0.02); neuroendocrine: 56 ± 6.3mm versus 51 ± 5.8mm (p = 0.01) (Table 3 and Figure 4).

In addition, a subgroup analysis was performed for largest tumor diameter comparison between DP-CBCT and CE-CT for patients (n = 18) with a time interval less than 30 days between the DP-CBCT and CE-CT showing significantly larger diameter in DP-CBCT (Mean 53.6 mm, SD 22.89) compared to CE-CT (Mean 45.2 mm, SD 19.23) (P = 0.0015). Mean difference with 95% CI: 10.6111 (–4.68; –16.54).

### Tumor contrast enhancement

**Figure 5:**
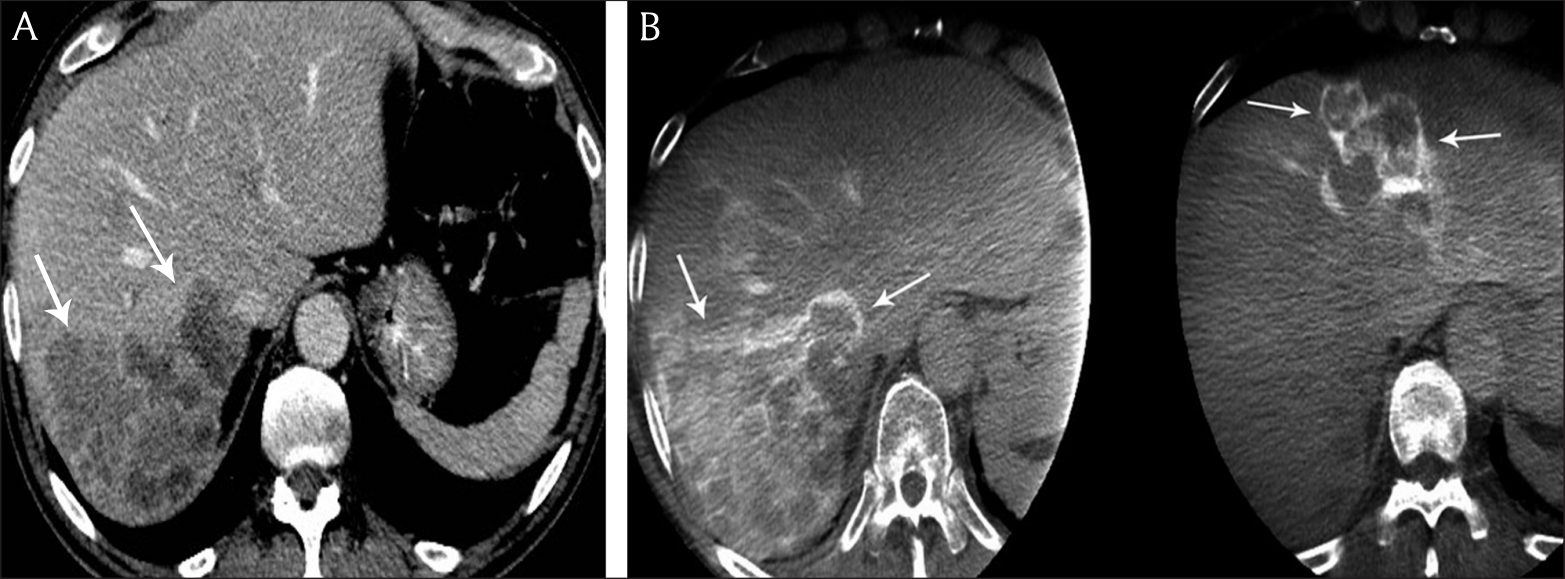
**A)** Contrast-enhanced computed tomography and **B)** cone beam computed tomography in a 68-year-old man presenting with bilobar colon cancer liver metastases. Contrast-enhancement of the peripheral tumoral rim is hyperdense to the residual liver parenchyma on cone beam computed tomography versus contrast-enhanced computed tomography.

The density and the rim enhancement of the liver metastases on both CE-CT and DP-CBCT images are summarized in Table 3 (Figure 5).

## Discussion

This study demonstrates that the use of DP-CBCT during catheter-directed liver interventions substantially may improve the detectability of the number of metastases, irrespective of the nature of the primary tumor, compared to pre-interventional, intravenous CE-CT. This high detection accuracy of liver metastases with DP-CBCT was also confirmed in a retrospective comparison by Schernthaner et al., who found that almost 40% of all metastases detected by DP-CBCT could not be identified by DSA and that all metastases detected by DP-CBCT were also visualized by pre-interventional contrast-enhanced magnetic resonance imaging (MRI) [22]. An important observation is that the DP-CBCT technique reported by these authors involved a sub-lobar injection protocol targeted to the lesions identified on the pre-interventional CE-MRI, which might represent a bias as only a small part of the liver was analyzed, which included lesions identified on MR imaging. It also may prevent a more thorough comparison of the detection accuracy among the different modalities. The diagnostic performance of DP-CBCT has been studied extensively in primary liver tumors, particularly hepatocellular carcinoma (HCC). It has been demonstrated that DP-CBCT is as accurate as CE-CT for the detection of HCC-lesions when the contrast medium is injected from the proper or common hepatic artery [17] and more accurate than CE-CT for lesions < 1m diameter if contrast injection is performed at the segmental level of the hepatic artery [15].

An important finding of our analysis is that the maximum diameter of the metastases visualized on CE-CT might be underestimated compared to findings on DP-CBCT. It is still unclear if the larger diameter on the CBCT-images is related to the enhancing rim around the hypodense, necrotic center of the metastasis, which is not always detected on venous phase CE-CT. This peripheral, enhancing rim, visualized during intra-arterial injection of contrast medium can be considered as a ‘corona enhancement’ as seen in the late arterial phase of DP-CBCT in HCC lesions [20]. However, it remains unclear if the larger diameter of the liver metastases on DP-CBCT is related to a larger tumor burden or to an inflammatory rim around the lesion as no pathological proof was available for this study. In addition to this observation, we were also able to demonstrate that a substantial number of metastases are considered as hypodense on venous phase CE-CT, but clearly present a hypervascular peripheral rim on DP-CBCT, which makes these metastases even better candidates for transarterial therapies like chemo- or radioembolization [23].

From a clinical, therapeutic perspective, these observations might have a pronounced effect on treatment planning of patient-tailored liver interventions. First, accurate analysis of the number, location, and volume of the metastases based on dual phase CBCT might impact the interventional strategy, such as adapting the positioning of the tip of the microcatheter prior to infusion of the chemoembolic mixture or radiolabeled microspheres, as also supported by Louie et al. [24], who studied the changes in treatment approach based on the use of CBCT versus DSA and 99Tc MAA-scintigraphy alone prior to yttrium-90 radioembolization. Additionally, a more accurate estimation of the tumor load might also affect the total dose calculation of radiolabeled microspheres in case of radioembolization [25], or in extreme cases, might lead to the abortion of the whole procedure in case of too high tumor load in combination with a borderline residual liver function.

The present study has some limitations. First, the patient sample size is small and only metastases of three different origins (colorectal cancer, breast cancer and neuroendocrine tumors) were included. However, the number of patients with metastases referred to catheter-directed liver interventions is still low owing to the paucity of data [4,5,7] to support these referrals and not all tumors are primarily metastasizing to the liver. Second, in this study DP-CBCT was compared to venous phase CE-CT, as this is the routine cross-sectional imaging method of choice for the follow-up of patients with liver metastases at our institution. We did not perform additional triphasic MDCT or contrast-enhanced MRI before catheter-directed liver intervention. Comparison of CE-MRI versus dual phase CBCT might show equal accuracy as indicated by Schernthaner et al. [22], although more studies on the topic are recommended. Third, this study focused on the retrospective comparison of two different imaging modalities to detect liver metastases, and no assessment was made on the potential clinical or therapeutic impact of these outcomes. Fourth, the time interval between CE-CT and DP-CBCT was variable, with a mean time interval of 28 days. Potentially a growth of metastases in this time interval is not excluded and might result in larger volumes when performing DP-CBCT. Last, we did not analyze the clinical outcome or overall survival after liver interventions with or without the addition of DP-CBCT [16].

In conclusion, this study demonstrates that while DP-CBCT has similar image quality to CE-CT, its diagnostic performance during catheter-directed liver interventions with regard to number, maximal diameter, and pattern of contrast enhancement of liver metastases from different origins is different to CE-CT. The additional information has the potential to alter the type and magnitude of dose per interventional strategy and thereby affect the clinical outcome of the patient. Prospective, multicenter trials with patients with the same type of cancer, treated with the same interventional modality, will confirm these results to improve the interventional treatment algorithm of secondary liver tumors.
